# Virulence-related metabolism is activated in *Botrytis cinerea* mostly in the interaction with tolerant green grapes that remain largely unaffected in contrast with susceptible green grapes

**DOI:** 10.1093/hr/uhac217

**Published:** 2022-09-21

**Authors:** Flávio Soares, Diana Pimentel, Alexander Erban, Catarina Neves, Pedro Reis, Marcelo Pereira, Cecilia Rego, Margarida Gama-Carvalho, Joachim Kopka, Ana Margarida Fortes

**Affiliations:** BioISI - Biosystems and Integrative Sciences Institute, Faculty of Sciences, University of Lisbon, Campo Grande, 1749-016 Lisboa, Portugal; BioISI - Biosystems and Integrative Sciences Institute, Faculty of Sciences, University of Lisbon, Campo Grande, 1749-016 Lisboa, Portugal; Max-Planck-Institut für Molekulare Pflanzenphysiologie, 14476 Potsdam-Golm, Germany; BioISI - Biosystems and Integrative Sciences Institute, Faculty of Sciences, University of Lisbon, Campo Grande, 1749-016 Lisboa, Portugal; LEAF—Linking Landscape, Environment, Agriculture and Food-Research Center, Associated Laboratory TERRA, Instituto Superior de Agronomia, Universidade de Lisboa, Tapada da Ajuda, 1349-017 Lisbon, Portugal; BioISI - Biosystems and Integrative Sciences Institute, Faculty of Sciences, University of Lisbon, Campo Grande, 1749-016 Lisboa, Portugal; LEAF—Linking Landscape, Environment, Agriculture and Food-Research Center, Associated Laboratory TERRA, Instituto Superior de Agronomia, Universidade de Lisboa, Tapada da Ajuda, 1349-017 Lisbon, Portugal; BioISI - Biosystems and Integrative Sciences Institute, Faculty of Sciences, University of Lisbon, Campo Grande, 1749-016 Lisboa, Portugal; Max-Planck-Institut für Molekulare Pflanzenphysiologie, 14476 Potsdam-Golm, Germany; BioISI - Biosystems and Integrative Sciences Institute, Faculty of Sciences, University of Lisbon, Campo Grande, 1749-016 Lisboa, Portugal

## Abstract

*Botrytis cinerea* is responsible for the gray mold disease, severely affecting *Vitis vinifera* grapevine and hundreds of other economically important crops. However, many mechanisms of this fruit-pathogen interaction remain unknown. The combined analysis of the transcriptome and metabolome of green fruits infected with *B. cinerea* from susceptible and tolerant genotypes was never performed in any fleshy fruit, mostly because green fruits are widely accepted to be resistant to this fungus. In this work, peppercorn-sized fruits were infected in the field or mock-treated, and berries were collected at green (EL32) stage from a susceptible (Trincadeira) and a tolerant (Syrah) variety. RNAseq and GC–MS data suggested that Syrah exhibited a pre-activated/basal defense relying on specific signaling pathways, hormonal regulation, namely jasmonate and ethylene metabolisms, and linked to phenylpropanoid metabolism. In addition, putative defensive metabolites such as shikimic, ursolic/ oleanolic, and *trans*-4-hydroxy cinnamic acids, and epigallocatechin were more abundant in Syrah than Trincadeira before infection. On the other hand, Trincadeira underwent relevant metabolic reprogramming upon infection but was unable to contain disease progression. RNA-seq analysis of the fungus *in planta* revealed an opposite scenario with higher gene expression activity within *B. cinerea* during infection of the tolerant cultivar and less activity in infected Trincadeira berries. The results suggested an activated virulence state during interaction with the tolerant cultivar without visible disease symptoms. Together, this study brings novel insights related to early infection strategies of *B. cinerea* and the green berry defense against necrotrophic fungi.

## Introduction

Grapevine is one of the most valuable and cultivated crops throughout the world. Most of the cultivars used for wine production are *V. vinifera* species which were selected due to its organoleptic characteristics. However, this species is highly susceptible to biotic stresses, mostly caused by oomycetes or fungi such as *Botrytis cinerea* [1, 2]. *B. cinerea* is a widespread, filamentous, and necrotrophic fungus that infects more than 200 plant species, leading to serious economic losses every year [3, 4]. This pathogen causes grey mold (bunch rot), one of the most severe diseases in grapevines, affecting the yield and quality of production worldwide. As a result, frequent applications of fungicides are needed to protect vineyards, with tremendous economic implications and compromising environmental sustainability [5].

The plant innate immune system (PIIS), a multi-layer and tightly regulated signal transduction component, triggers proteins and metabolites with a defensive role against different pathogens [6]. The PIIS is composed of the pathogen-associated molecular pattern (PAMP)-triggered immunity (PTI) and effector-triggered immunity (ETI) [7]. PAMPs are recognized by the plant pattern recognition receptors (PPRs), a system of receptor-like kinases or receptor-like proteins, crucial for cell-to-cell communication and extracellular signal sensing [8]. Different examples of PAMPs have been described, such as flg22 (bacterial flagellin), elf18 (bacterial elongation factor-Tu) and, regarding fungi, cell wall polysaccharides, chitin, β-glucans, and ergosterol [9]. For example, the endopolygalacturonases from *Botrytis* are recognized by the receptor-like protein RBPG1 in Arabidopsis and induce the PTI response, which is not strong but is broad-spectrum immune response [10]. Effector-triggered immunity is the second level of pathogen recognition and requires the perception of pathogen-specific effectors, recognized by plant R proteins and leading to a rapid and robust response [11, 12]. Pathogen recognition by the PTI/ETI systems is followed by a complex signaling network that regulates gene expression and the activation of several downstream defense-related pathways, such as the induction of reactive oxygen species (ROS), cell wall modifications to limit fungal growth, or/and the activation of calcium signaling and MAPK cascades resulting in the expression of many defense-related genes and production of secondary metabolites such as phytoalexins [13–15].

The aforementioned plant defenses are shaped by several phytohormones, including salicylic acid (SA), classically associated with resistance against biotrophic pathogens; and jasmonic acid (JA) and ethylene (ET) linked with resistance to necrotrophic fungi, including *B. cinerea* [15, 16]*.* Nevertheless*,* there are several exceptions to this, and the participation of growth and stress-related hormones such as gibberellic and abscisic acid, auxin, and brassinosteroids in plant defense activity has also been described [4, 6]. On the other hand, necrotrophic pathogens have evolved complex strategies to subdue the host immune system. *B. cinerea* can infect grapevine by mycelium penetration through stomata and wounds or by conidia early invasion, infecting mainly the flower receptacle area and remaining quiescent until berry maturation [17, 18]. In favorable conditions, the conidium develops the appressorium, a specialized infective structure that secretes several phytotoxins and lytic enzymes and promotes an oxidative burst that facilitates host colonization [19]. Nevertheless, in the early stages of infection and before the necrotrophic phase, the fungus can exhibit a short biotrophic behavior that allows the accumulation of biomass and establishment inside the host [20].

Recent studies have been trying to clarify the transcriptome landscape behind *B. cinerea* virulence in different species, such as cucumber and *A. thaliana* leaves [6, 21], kiwifruit [22], tomato fruit, and others [23]. Moreover, the fungus transcriptome during infection of grapes (cv. Marselan) was accessed at harvesting stage by microarrays [24], and by RNAseq in cv. Pinot Noir at flowering (EL25/26) [18] and ripening stage [24]. Haile and colleagues [25] also reported the *in planta B. cinerea* transcriptome during fungus quiescent state in green hard berries [25]. Nevertheless, all these studies addressed only one cultivar and the mechanisms behind the unusual infection of green berries remain undiscovered. Moreover, the complex host/*B. cinerea* pathosystem continues to stand poorly understood and more studies are needed, especially due to the extremely plastic transcriptomes of both organisms, which are influenced in a bidirectional manner [6].

Grape clusters can be naturally infected by *B. cinerea* before bloom and after *veraison* with increasing susceptibility from *veraison* to ripening. Between flower and *veraison*, grape berries are known to be naturally resistant to *B. cinerea* infection [26, 27]. However, recent studies showed that certain varieties may become infected at green stage when artificial in-field infections are performed [28]. Such is the case of Trincadeira, a very important Portuguese cultivar, which is extremely susceptible to *B. cinerea*. Additionally, recent studies focused on hormonal metabolism indicated that tolerance against this necrotrophic fungus is mostly based on basal defense, whereas susceptibility is due to delayed defensive responses [16].

In the present work, we compared for the first time the transcriptome and metabolome associated with green hard berries infected with *B. cinerea* from susceptible (Trincadeira) and tolerant (Syrah) cultivars, bringing innovative insights regarding the early regulatory mechanisms involved in tolerance/susceptibility*.* Moreover, *in planta* associated pathogen transcriptomes were analyzed in both grapevine cultivars, disclosing the dynamics of early infection in opposite host scenarios.

## Results

### Metabolic alterations stimulated *B. cinerea* infection in both Syrah and Trincadeira grape berries

**Figure 1:**
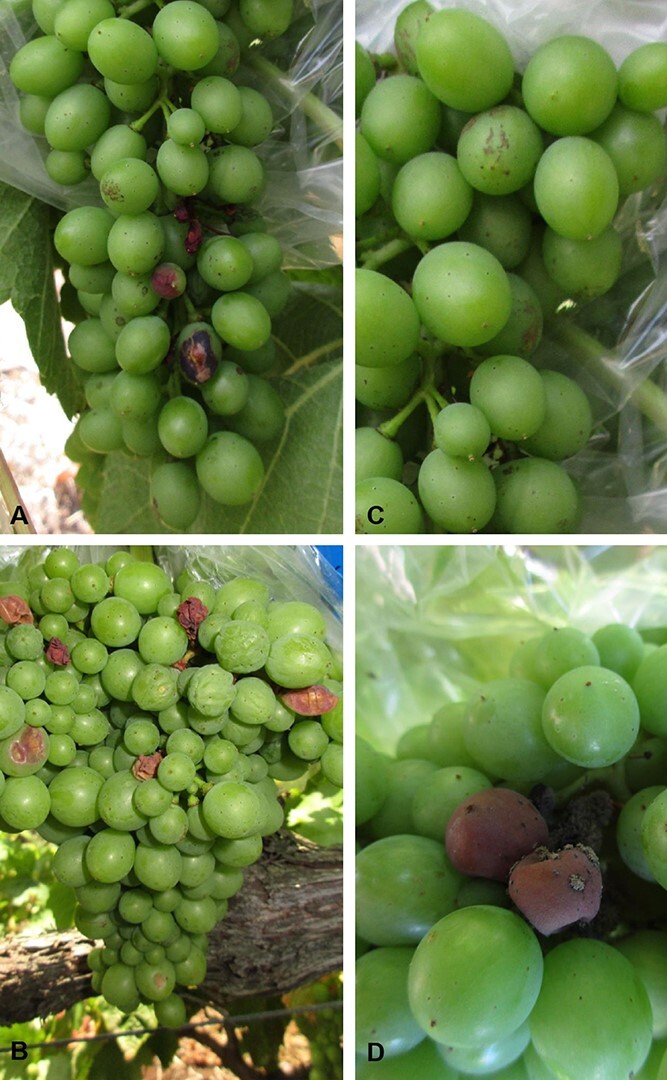
Clusters of *Vitis vinifera cv.* (A) Syrah and (B) Trincadeira grapes naturally infected with grey mold (*Botrytis cinerea)* at green developmental stage (EL32). (C) Magnification of Syrah clusters. (D) Magnification of Trincadeira clusters: fungal sporulation was observed in infected Trincadeira clusters.

Green berries are widely recognized as resistant to *Botrytis cinerea* infection. Notwithstanding, our previous work has shown that green berries of certain cultivars may exhibit heavy symptoms of infection under proper humidity and temperature condition [26, 28, 29]. In the present work, healthy and infected berries from Trincadeira and Syrah were sampled at green stage (EL32) according to the modified E–L system [30]. Trincadeira extreme susceptibility is thought to be due to high cluster compactness and high growth vigour that creates a low temperature and high humidity microclimate that favours *B. cinerea* infection [28].The visual analysis showed that green Trincadeira berries already presented a high level of *B. cinerea* infection and, in contrast, Syrah showed only mild symptoms with no fungal sporulation being observed (Fig. 1). This was previously confirmed by us for the same samples by qPCR using primers for *B. cinerea Polygalacturonase* (*BcPG1*) [16]. This data indicated that the percentage of infection was ~16X fold higher in Trincadeira at green stage than in Syrah. Additionally, though at ripe stage infection symptoms could be observed in this Syrah cultivar they were still less strong than in Trincadeira [16].

**Figure 2:**
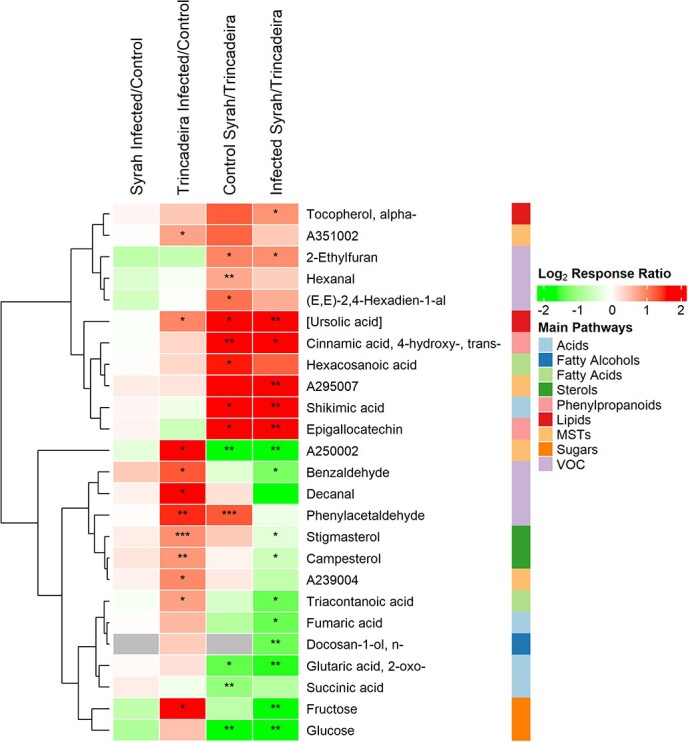
Infection- and cultivar-responsive metabolites from *Botrytis cinerea* infected and control berries of *Vitis vinifera* cv. Trincadeira and cv. Syrah at the green developmental stage (EL32). First two columns compare each infected cultivar with its control. Third column compares Syrah control with Trincadeira control. Fourth column compares infected Syrah with infected Trincadeira. Soluble and volatile metabolites that were significantly changed in at least one comparison (response ratio ≥ 1.5 and p-value ≤0.05) with main pathways tagged. Response ratios were log_2_-transformed and hierarchically clustered using Euclidean distance and complete linkage. Asterisks present statistical significance comparing to the control (^*^ p-value ≤0.05; ^**^ p-value ≤0.01; ^***^ p-value ≤0.001).

To gather insights on how grapes’ metabolism was affected by the infection, GC-EI-TOF/MS was used for the relative quantifications of sugars, organic acids, phenylpropanoids, and other soluble metabolites. Profiling of volatile metabolites was achieved using a GC-EI/QUAD-MS (Supplementary Table S1). Principal component analysis (PCA) was performed with normalized responses (Supplementary Table S1); PC1 and PC2 accounted for 47.70% of the total variability (Supplementary Fig. S1) and discriminated samples based on cultivar and infection status, respectively. Within each cultivar, PC2 only established a clear separation between Trincadeira samples, as all Syrah samples were plotted together, revealing a similar metabolic content among control and infected berries. Twenty-five metabolites (23.4% of all detected species) displayed statistically significant differences in abundance between cultivars or between control and infected samples of the same cultivar (Fig. 2 and Supplementary Table S1).

Several differences were observed at basal levels when comparing tolerant and susceptible cultivars (Fig. 2). Specifically, the phenylpropanoids epigallocatechin and *trans*-4-hydroxycinnamic acid were constitutively present in a larger amount in Syrah, together with shikimic acid, a precursor of phenylpropanoids; those compounds accumulated at higher levels in Syrah than in Trincadeira upon infection. Moreover, the volatile organic compounds (VOCs) 2-ethylfuran, hexanal, (E, E)-2,4-Hexadien-1-al, and phenylacetaldehyde were detected in higher amounts in control Syrah than in control Trincadeira.

Regarding fatty acids, Syrah showed superior basal levels of hexacosanoic acid (C26) than Trincadeira (Fig. 2 and Supplementary Table S1). Very-long-chain fatty acids are required for the biosynthesis of the plant cuticle, generation of sphingolipids and have been associated with plant defense [31]. Syrah also presented higher levels of a compound from the ursolic/ oleanolic acid family than Trincadeira in both basal and under infection conditions, and, in Trincadeira, this lipid increased in response to *B. cinerea* infection (Supplemental Table S1).

**Figure 3:**
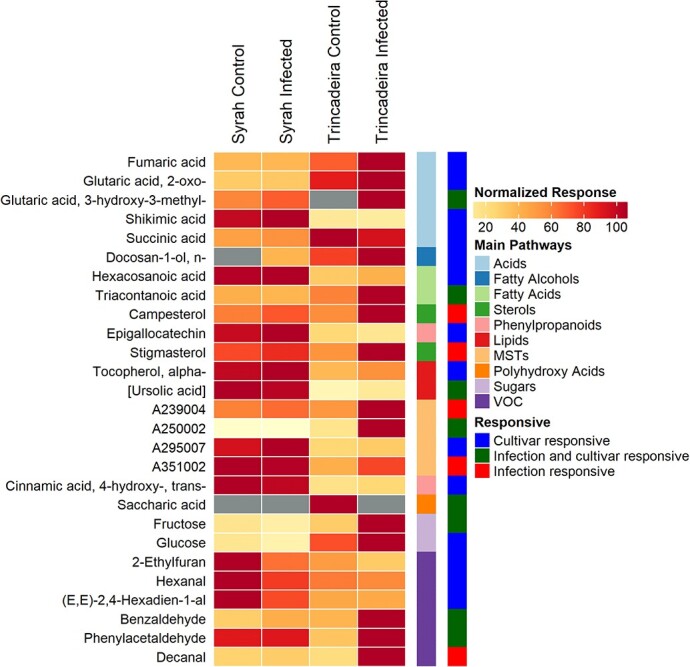
Putative positive metabolic markers of *B. cinerea* infection at green stage (EL32) of development of *Vitis vinifera* cv. Trincadeira and cv. Syrah. Metabolites presented were either significantly increased after infection at one or both cultivars (response ratio ≥ 1.5 and p-value ≤0.05) or only identified in infected berries. Square brackets indicate metabolites that were classified only by mass spectral match and grey boxes metabolites that were not detectable.

**Figure 4:**
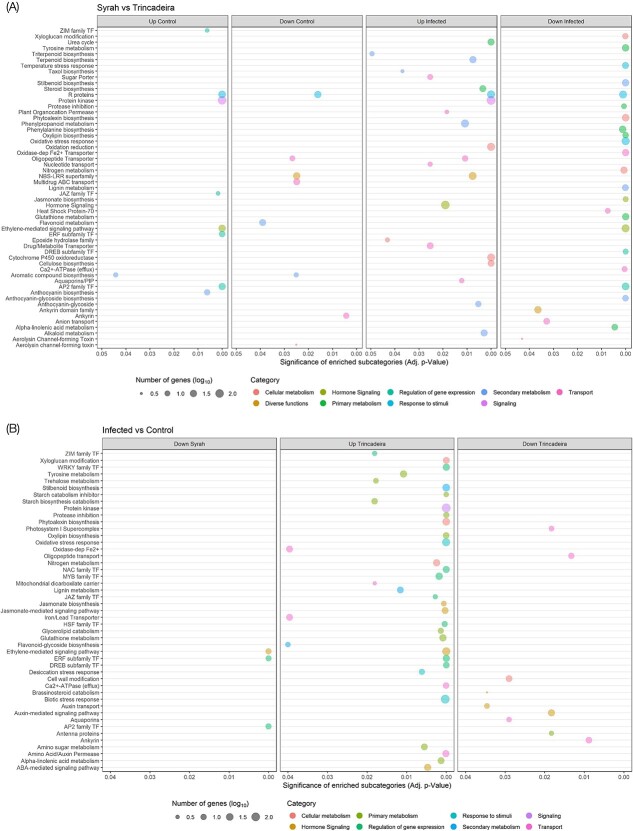
Enriched functional subcategories (A) in Syrah-responsive transcripts (Syrah vs Trincadeira) and (B) in *Botrytis cinerea* infection-responsive transcripts (Infection vs Control) (adjusted p-value ≤0.05). Circles’ size represents the number of genes (log_10_). A complete dataset in Supplemental Table S5 and S6.

On the other hand, Trincadeira showed higher basal levels of glucose, succinic acid, and 2-oxoglutaric acid than Syrah; additionally, 2-oxoglutaric acid increased in Trincadeira upon infection together with fructose, benzaldehyde, and the sterols stigmasterol and campesterol (Fig. 2 and Supplementary Table S1). These compounds reached higher content in this susceptible cultivar than in Syrah, infected samples. Campesterol is a crucial precursor of brassinolide, which plays important role in several signaling pathways to reduce biotic stress damage [32]. Phenylacetaldehyde and decanal, putative markers of ripening in grape berries, were increased in Trincadeira upon infection, indicating an acceleration of ripening in susceptible Trincadeira promoted by the fungus *B. cinerea* as previously reported [28]*.*

Finally, when comparing infected berries, triacontanoic acid, fumaric acid, and the fatty alcohol n-docosan1-ol were detected in higher amounts in Trincadeira, whereas the antioxidant α-tocopherol was increased in Syrah (Fig. 2 and Supplementary Table S1).

In general, *B. cinerea* infection had little influence on the Syrah metabolome at the green stage. On the other hand, ten metabolites were identified as putative markers of Trincadeira infection, revealing an early metabolic reprogramming upon infection in this susceptible cultivar (Fig. 3 and Supplementary Table S1). Moreover, eight metabolites were more accumulated in Syrah at basal level and might thus be putative markers of tolerance (Figs. 2, 3 and Supplementary Table S1).

### RNAseq and functional enrichment analysis indicate a strong transcriptional reprogramming in Trincadeira under infection that was not observed in Syrah

Transcriptional profiling was performed using three biological replicates of control and infected berries of each cultivar. Supplementary Table S2 shows the parsed reads and reads mapped to the predicted transcriptomes, both for *B. cinerea* and *V. vinifera*. In our study, the average number of reads uniquely mapped to the grapevine genome was higher in Trincadeira infected samples than in Syrah (19 272 633 and 10 553 075 reads, respectively). On the other hand, *B. cinerea* average reads *in planta* were higher in the tolerant infected cultivar (6021 reads, representing 0.057% of the total) than in the susceptible cultivar (2570, representing 0.013% of total reads) (Supplementary Table S2).

The expression of 26 110 different grape genes (87.11% of the total predicted grape genes [33]) and 5478 different *B. cinerea* genes (44.83% of the total predicted *B. cinerea* genes [34]) was detected across all samples. Multi-dimensional scaling (MDS) plot of all normalized grape gene counts separated the data into three groups (Supplementary Fig. S2). Similar to the metabolomic results, Syrah samples clustered together independently of the infection status whereas for Trincadeira the MDS plot discriminated infected from control samples.

Differences in gene expression between Trincadeira and Syrah were analyzed comparing the constitutive and under infection transcriptome of both cultivars, and individually for each variety (Infected vs Control) (Supplementary Table S3). The number of differentially expressed genes (DEGs) is reported in Supplementary Table S4. A total of 10 555 grape genes were differentially expressed (FDR ≤ 0.05 and log2 |FC| > 1.0) due to the cultivar (7576 genes) or/and infection status (2979 genes). The Venn diagram illustrates that from the DEGs detected when comparing both infected cultivars, most of them were already detected when comparing the cultivars before infection, implying that the majority of differences are unrelated to specific responses to infection (Supplementary Fig. 3A). The remaining DEGs are likely explained by changes in the Trincadeira transcriptome since there were only 22 DEGs detected in Syrah upon infection (Supplementary Fig. 3B). These results suggest that *B. cinerea* presence had limited influence on the Syrah transcriptome at the green stage of grape development.

Key biological processes activated or repressed in cultivars (Fig. 4A and Supplementary Table S6) or due to infection (Fig. 4B and Supplementary Table S5) were determined by enrichment analyses of functional categories (P-value ≤0.05) using FatiGO [35]. Several functional classes were upregulated in Trincadeira after infection: signaling, carbohydrate-related (including trehalose, starch, and amino sugars metabolism), secondary metabolism (lignin metabolism, stilbenoid, and flavonoid biosynthesis), cell wall-related (xyloglucan modification), stress response (such as biotic and desiccation, oxidative stress), phytoalexin biosynthesis, hormone signaling (mainly ethylene and jasmonate signaling) and lipid metabolism (oxylipin biosynthesis, glycerolipid, and α-linolenic acid metabolism). Moreover, several families of transcription factors were also found to be enriched in the set of genes that was upregulated in Trincadeira under infection (ZIM, WRKY, NAC, MYB, JAZ, ERF, and others). In contrast, an enrichment of the functional classes related to cell wall modification and photosynthesis was observed among the genes downregulated in Trincadeira infected samples (Fig. 4B).

Finally, only three functional categories were enriched in Syrah infected berries. These include, aquaporins, ethylene-mediated signaling pathway, and the ERF subfamily of transcription factors, all of them among the downregulated genes (Fig. 4B and Supplementary Table S5).

#### Genes involved in signaling pathways associated with defense are constitutively highly expressed in Syrah whereas in Trincadeira they are activated in response to infection

Categories of genes encoding R proteins, protein kinases, proteins involved in calcium signaling, ET-mediated signaling, and ZIM, JAZ, and AP2/ERF families of transcription factors are enriched in Syrah when comparing both cultivars at basal level. Expression of genes belonging to these categories was activated in Trincadeira only in response to *B. cinerea* infection (Fig. 4B and Supplementary Table S5). In detail, *B. cinerea* infection led to an increase of calcium signaling in Trincadeira, as suggested by the upregulation of many genes involved in calcium-sensing and signaling. Most of those genes were already highly expressed in Syrah when compared to Trincadeira before infection (e.g. calmodulin, calmodulin-binding proteins, and calcium-transporting ATPases) (Table 1). The same holds for the protein kinase functional category, with several up-regulated genes putatively encoding for protein kinases, receptor serine/threonine kinases, and for leucine-rich repeat receptor kinases, which appear to play central roles in signaling during pathogen recognition and plant defense mechanisms (Table 1 and Supplementary Table S3) [36, 37]. Moreover, genes coding for GRAS transcription factors which have been associated with grapevine response to biotic stress [38] were upregulated in Trincadeira under infection and constitutively upregulated in Syrah (Table 1 and Supplementary Table S3).

Finally, the activation of defense-related genes in plants has been associated with different phytohormones, with JA and ET being essential for plant innate immune system against necrotrophic fungi [4, 39]. Among upregulated genes in Syrah before infection were those involved in ET synthesis and jasmonates´ signaling; these genes were upregulated in Trincadeira after infection (Table 1 and Supplementary Table S3). Previous hormonal profiling performed in the same samples revealed the importance of jasmonates among other hormones in response to *B. cinerea* during early stages of ripening [16]. This study also validated by qPCR the present RNAseq data regarding hormonal metabolism.

#### Basal and under infection primary and secondary metabolism are strikingly different in between tolerant and susceptible cultivars

Functional enrichment analysis revealed a broad transcriptional contrast between primary and secondary metabolisms of the two cultivars before and under infection. Primary metabolism, in general, was activated in Trincadeira under infection (enrichment in amino sugar, tyrosine, nitrogen, and trehalose metabolisms, glycerolipid catabolism, and α-linolenic acid metabolism, as well as oxylipin biosynthesis and proteinase inhibitors functional classes) (Table 1 and Supplementary Table S3). Moreover, in Trincadeira, the infection led to the upregulation of genes involved in oxidative stress response, desiccation, temperature, and biotic stress response among others (Table 1 and Supplementary Table S3). On the other hand, urea cycle, photosynthesis, and steroid biosynthesis seem to be inhibited in Trincadeira, when comparing both infected cultivars (Table 1 and Supplementary Table S3). Interestingly, transcripts involved in the biosynthesis of the antioxidant α-tocopherol were upregulated in Syrah at basal level and downregulated in Trincadeira upon infection, in agreement with metabolomics data (Table 1 and Supplementary Table S3).

Concerning the cell wall metabolism, many genes encoding laccases, pectinesterases, and xyloglucan modifications were upregulated in Trincadeira under infection (Table 1). On the other hand, cellulose biosynthesis seems to be activated in Syrah, as suggested by the upregulation of several cellulose synthases when comparing both cultivars before and after infection. Furthermore, carbohydrate metabolism was also affected in Trincadeira by *B. cinerea*, mainly due to the upregulation of several genes encoding *α-* and *β-*amylases and differently expressed genes coding sugar transporters (Table 1 and Supplementary Table S3).

Regarding secondary metabolism, many genes related to alkaloid metabolism and biosynthesis of taxol, terpenoids, and triterpenoids were downregulated in Trincadeira upon infection (Table 1 and Supplementary Table S3). On the other hand, anthocyanin biosynthesis was activated in Syrah pre-infection but triggered by the fungus in Trincadeira, together with stilbenoid biosynthesis. Finally, phenylpropanoid metabolism was enriched in Syrah when comparing both infected cultivars. (Table 1 and Supplementary Table S3). In general, the data showed several genes involved in secondary metabolism already activated in Syrah at basal level and downregulated in Trincadeira upon *B. cinerea* infection.

**Table 1:** Selected grapevine differentially expressed genes in susceptible and tolerant cultivars (FDR ≤ 0.05 and log2 |FC| > 1.5). Complete dataset in Supplementary Table S3

Unique ID	log Fold change				Functional annotation (Grimplet et al. 2012)
	Syrah Infected/ Syrah Control	Trincadeira Infected/ Trincadeira Control	Syrah Control/ Trincadeira Control	Syrah Infected/ Trincadeira Infected	
*Biotic stress response and secondary metabolism*					
VIT_00s0266g00070		−3.34			Linalool synthase
VIT_01s0010g02930		4.14	3.16	−2.61	Calmodulin
VIT_03s0038g04390		5.89		−3.83	Dehydrin 1
VIT_04s0210g00120			6.66	5.70	Strictosidine synthase
VIT_05s0020g03200			2.04	3.24	Spermine synthase
VIT_05s0077g01690		7.06	3.88	−2.53	Pathogenesis protein 10 [*Vitis vinifera*]
VIT_06s0004g05310		−1.95	2.38	4.60	Tropinone reductase
VIT_06s0004g07650		−1.51		2.52	Taxadien-5-alpha-ol-O-acetyltransferase
VIT_09s0002g00220		2.76	2.35	−1.68	Avr9/Cf-9 rapidly elicited protein 132
VIT_10s0003g03650			8.83	8.73	Beta-amyrin synthase
			7.70	5.19	Beta-amyrin synthase
VIT_11s0016g05010		4.03		−4.72	Lactoylglutathione lyase
VIT_12s0034g00130		3.76	1.54	−3.27	Anthocyanidin 3-O-glucosyltransferase
VIT_12s0035g01000		10.3		−10.1	Serine protease inhibitor, serine-type
VIT_13s0064g00340			6.52	6.82	Cinnamyl alcohol dehydrogenase
VIT_14s0068g01920		2.56			Peroxidase
VIT_14s0081g00770			7.33	7.91	R protein disease resistance protein
VIT_16s0039g01280		3.95		−4.91	Phenylalanin ammonia-lyase [*V. vinifera*]
VIT_16s0039g01870		4.47	3.58	−2.82	Protein kinase
VIT_16s0100g00090		4.88		−2.72	Cationic peroxidase
VIT_16s0100g01070		6.48		−6.66	Resveratrol synthase [*V. vinifera*]
VIT_16s0100g01190		6.89		−6.09	Stilbene synthase [*V. vinifera*]
VIT_18s0041g00920		4.96	7.81	3.40	UDP-glucose: anthocyanidin 5,3-O-glucosyltransferase
VIT_18s0117g00370			10.7	10.8	R protein L6
*Oxidative stress*					
VIT_04s0008g03600			1.68	3.77	Tocopherol cyclase
VIT_04s0079g00690		11.96		−11.79	Glutathione S-transferase 26
VIT_08s0040g00920		4.96	5.89	3.93	Glutathione S-transferase 25
VIT_10s0003g00390		4.04	2.64	−2.25	Glutaredoxin
VIT_16s0039g01410			2.12	2.81	Tocopherol O-methyltransferase
*Signaling, Transcription factors and Kinases*					
VIT_00s0425g00030			7.07	3.77	Receptor serine/threonine kinase
VIT_00s0463g00020		1.78	1.35	−1.59	Scarecrow transcription factor 5 (SCL5)
VIT_02s0033g00390		7.92	−2.55	−10.6	Myb domain protein 113
VIT_06s0004g04990			4.81		Scarecrow transcription factor 14 (SCL14)
VIT_06s0061g01400		5.16			CBF transcription factor [*V. vinifera*]
VIT_07s0031g01710		4.22	2.56		WRKY DNA-binding protein 51
VIT_08s0007g03630			2.10	2.01	Calmodulin binding protein
VIT_08s0007g08750		3.24			Heat shock transcription factor B3
VIT_08s0032g01220		3.16	2.49	−2.26	Calcium dependent protein kinase 1
VIT_12s0142g00800			2.49	5.97	Leucine-rich repeat protein kinase
VIT_19s0014g04040		6.95	9.01	2.67	S-receptor protein kinase
VIT_19s0014g04940		2.22	1.55	−1.63	Chitin-inducible gibberellin-responsive protein 1
*Hormonal metabolism*					
VIT_00s0253g00150		2.57	3.35		Methyl jasmonate esterase
VIT_03s0063g01820		4.81		−7.50	AOS (allene oxide synthase)
VIT_04s0008g02230		2.87	3.02	−2.27	AP2 domain-containing transcription factor ORA47
VIT_04s0008g05760		3.17	2.68		WRKY DNA-binding protein 18
VIT_05s0049g00510		2.33	−1.97	−3.76	Ethylene response factor ERF1
VIT_09s0002g09140			−3.66	−3.11	Ethylene-responsive transcription factor ERF003
VIT_10s0003g00590		4.31	3.77	−1.46	Ethylene-responsive transcription factor ERF091
VIT_10s0003g03800		2.95	3.02	−1.51	Jasmonate ZIM domain-containing protein 8
VIT_11s0016g00660		5.56	3.89	−5.03	DREB sub A-5 of ERF/AP2 transcription factor
VIT_11s0016g00670		2.56	2.27		AP2 domain-containing transcription factor
VIT_11s0016g00710		2.13	1.80		Jasmonate ZIM-domain protein 1
VIT_15s0046g02220		7.12	3.91	−4.11	ACC synthase
VIT_16s0013g00980	−1.59		2.60		Ethylene-responsive transcription factor ERF105
*Carbohydrate metabolism*					
VIT_00s0181g00180		−1.71			LHCB3 (light-harvesting chlorophyll binding protein 3)
VIT_02s0154g00110		3.18		−1.84	Trehalose-6-phosphate phosphatase (AtTPPA)
VIT_05s0020g03140		5.57		−3.53	Sugar transporter 13
VIT_05s0077g00840		3.60	−2.19	−6.23	Galactinol-raffinose galactosyltransferase
VIT_07s0005g01680		3.75		−3.45	Stachyose synthase
VIT_07s0005g02220		−1.80			LHCII type I CAB-1
VIT_14s0030g00220		−2.20		2.97	Sugar transporter ERD6-like 5
VIT_14s0030g00300			2.02	1.81	Sugar transporter ERD6-like 3
VIT_14s0060g00760		4.67		−4.73	Galactinol synthase
VIT_14s0066g00810		3.15		−3.62	Raffinose synthase
VIT_17s0000g01820		3.24		−1.51	Malate synthase, glyoxysomal
VIT_17s0119g00150		7.65	4.01	−5.12	Alpha-amylase/subtilisin inhibitor
*Cell wall metabolism*					
VIT_00s2620g00010		2.75		−1.60	Endo-1,4-beta-glucanase korrigan (KOR)
VIT_01s0127g00870		−1.72		2.76	Polygalacturonase JP630
VIT_01s0137g00240		−2.39			Pectate lyase
VIT_06s0009g02590		11.05		−11.48	Pectinesterase family
VIT_07s0005g01030				2.93	Cellulose synthase CSLD5
VIT_08s0007g08330		7.19		−7.61	Polygalacturonase PG1
VIT_08s0040g01340		7.20		−7.03	Cellulose synthase CSLA09
VIT_11s0052g01180		3.33	2.00	−2.49	Xyloglucan endotransglucosylase/hydrolase 23
VIT_12s0059g01010			5.66	6.27	Cellulose synthase CSLB04
VIT_18s0122g00690		9.65		−8.42	Laccase
Lipid metabolism					
VIT_04s0079g00790		2.52		−2.09	Acyl-CoA synthetases (Acyl-activating enzyme 11)
VIT_06s0004g01500		2.26		−3.88	Lipoxygenase (LOX2)
VIT_07s0005g01240		3.47	1.70	−2.40	Triacylglycerol lipase
VIT_07s0141g00060		3.68		−4.80	Beta-ketoacyl-CoA-synthase
VIT_09s0002g01080		2.33	2.31		Lipoxygenase
VIT_13s0067g01120		2.12		−1.63	Omega-3 fatty acid desaturase, chloroplast, temperature-sensitive (FAD8)
VIT_14s0066g01670		6.80		−6.63	Alpha-dioxygenase
VIT_16s0022g01120			6.73		Acyl-CoA oxidase ACX3
VIT_16s0022g01150			7.56		Acyl-CoA oxidase ACX3

#### Botrytis cinerea presents higher transcriptional reprogramming in the tolerant cultivar

Analysis of RNAseq showed that thousands of reads uniquely mapped to the fungus genome were detected in all infected samples (Supplementary Table S2). Few fungal reads were also detected in all control samples (Supplementary Table S2) confirming the natural and opportunistic presence of *B. cinerea* in the vineyards [40]. Considering in detail the number of *B. cinerea* genes expressed *in planta*, 531 different genes were Syrah-specific, 166 Trincadeira-specific, and 122 shared by both cultivars (Supplementary Table S7 and S8). PCA plot of all fungus normalized genes counts grouped all the control samples and showed a clear separation between infected cultivars, with the PC1 explaining 71.7% of the variability between control and infected samples (Supplementary Fig. S4).

**Figure 5:**
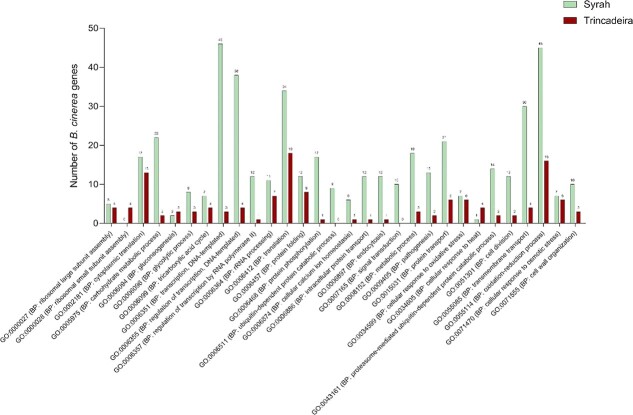
**–**Biological processes associated with fungal *in planta* expressed genes. Bar graph are the top 30 represented GOs sorted by number. Labels on the top of each bar indicate the number of genes that matched that particular enriched category. The complete dataset is presented in Supplemental Table S7.

As a necrotrophic pathogen, *B. cinerea* secrets a broad repertoire of virulence factors, triggering plant chlorosis and host cell death [41]. Several genes associated with virulence and growth were Syrah-specific (Table 2 and Supplementary Table S7). Fungal cell division appears to be promoted in the tolerant cultivar, as suggested by the expression of genes related to the cell cycle, cytoplasmic microtubule and actin cytoskeleton organization, and cellular amino acid biosynthetic process (Fig. 5 and Supplementary Table S7). Furthermore, the results showed a general activation of genes participating in ROS generation and/or oxidation–reduction processes, mainly in Syrah (Fig. 5 and Supplementary Table S7). In particular, the *BcNoxR (Bcin03g06840), a* major generator of ROS and essential for the development of sclerotia and full virulence [42] (Table 2 and Supplementary Table S7). The same holds true for genes involved in signaling pathways (fungal protein kinases, calcium signaling), protein regulation (e.g. translation, protein folding, and phosphorylation), and protein transport (Table 2 and Supplementary Table S7). Several *B. cinerea* ribosomal proteins involved in translation mechanisms were expressed in both cultivars. Moreover, were expressed mainly in Syrah genes with a putative role in transcription regulation, several transcription factors (e.g. MYB, BZIP, NOT, TFIID, SRF, SFP1, and CP2, and zinc fingers), and genes involved in chromatin structure and modification (Fig. 5, Table 2, and Supplementary Table S7).

**Table 2:** Selected *in planta* detected *Botrytis cinerea* transcripts. Genes were only considered present if normalized gene count (RPKM) was equal to zero in all three control replicates and higher than zero in all three infected replicates. Complete dataset in Supplementary Table S7

	RPKMs							
	Syrah Infected			Trincadeira Infected				
Unique ID	Inf2	Inf3	Inf4	Inf12	Inf35	Inf4	Gene Ensembl	Functional annotation
*Virulence and Growth*								
Bcin01g01520.1	1037	949.4	409.4					ER-derived vesicles protein ERV14
Bcin01g04560.1	175.1	240.5	622.3				*Bcphs1*	Bifunctional lycopene cyclase/phytoene synthase
Bcin01g05060.1	69.1	189.7	245.4					Aminopeptidase 2
Bcin01g08260.1				1542	1408	1372	*Bcrib4*	6,7-dimethyl-8-ribityllumazine synthase
Bcin02g01260.1	451.0	619.3	267.1					Pisatin demethylase
Bcin02g04870	822.0	376.3	324.5					Histone H1
Bcin02g08170.1	90.7	186.9	53.7				*Bmp1*	Mitogen-activated protein kinase
Bcin02g08280.1	100.4	137.9	237.9				*Bcswi1*	SWI/SNF chromatin-remodeling complex subunit sol1
Bcin03g00500.1	314.3	2158	372.3				*Bcspl1*	Protein SnodProt1
Bcin03g00750.1	982.1	224.8	387.7				*BczipA*	BZIP-type transcription factor MBZ1
Bcin03g01480.1	520.8	357.6	616.9				*Bcgpx3*	Glutathione peroxidase-like peroxiredoxin HYR1
Bcin03g03390.1	2136	2933	2024				*Bcsod1*	Superoxide dismutase [Cu-Zn]
Bcin03g03880.1	301.1	206.8	356.7				*Bcdoa1*	Ubiquitin homeostasis protein lub1
Bcin04g03690.1	165.8	227.8	392.9				*Bcvps1*	Vacuolar protein sorting-associated protein 1
Bcin04g05630.1	171.4	470.7	203.0				*Bcste7*	Dual specificity protein kinase FUZ7
Bcin05g00730.1	5988	2530	1909	2969	4521	11 448	*Bccat4*	Peroxisomal catalase
Bcin05g01450.1	1022	468.3	1009				*Bcprd2*	Dothistromin biosynthesis peroxidase dotB
Bcin05g05530.1	347.3	238.5	411.3				*Bcwcl2*	Cutinase gene palindrome-binding protein
Bcin05g06320.2				3019	2759	6270	*Bcp1*	Peptidyl-prolyl cis-trans isomerase, mitochondrial
Bcin05g07640.1	214.3	588.7	1015					Cyrochrome P450 monooxygenase cloA
Bcin06g02460.1	384.0	527.3	454.8				*Bcgst4*	Disulfide-bond oxidoreductase YghU
Bcin06g04460.1	651.9	1342	1158					Catechol 1,2-dioxygenase
Bcin07g03340.1	216.1	296.8	255.9				*Bcnma*	Pro-apoptotic serine protease NMA111
Bcin07g04950.1	226.6	311.2	536.9					Questin oxidase
Bcin08g03620.1	93.2	64.0	55.2					ABC transporter aclQ
Bcin09g03570.1	555.8	763.3	658.3				*Bcatp7*	ATP synthase subunit d, mitochondrial
Bcin09g06140.1	53.0	218.4	62.8				*Bcmcm1*	Transcription factor of morphogenesis MCM1
Bcin10g00740.1	422.1	579.6	499.9				*Bcgst1*	Glutathione S-transferase-like protein gedE
Bcin10g01180.1	1757	1689	624.5	1510	4139	1343	*Bcarb1*	ABC transporter ATP-binding protein ARB1
Bcin10g05640.1				1052	961.2	2808	*Bcbim1*	Microtubule integrity protein mal3
Bcin11g01720.1	825.0	679.8	1172.6				*Bcltf3*	Transcriptional regulator NRG1
Bcin11g05430.1	243.4	334.3	576.6					MFS transporter prlL
Bcin12g01370.1				587.6	268.4	522.9	*Bcmsb2*	Signaling mucin MSB2
Bcin14g01550.1	367.2	168.1	145.0				*Bcatg2*	Autophagy-related protein 2
Bcin15g02590.1	57.3	118.0	67.9				*Bac*	Adenylate cyclase
Bcin15g03610.1	146.5	201.2	173.6				*Bcg3*	Guanine nucleotide-binding protein alpha-3 subunit
Bcin16g03140.1	715.9	983.1	565.3				*Bccbf5*	Centromere/microtubule-binding protein cbf5
*Signalling*								
Bcin01g00930.1	340.6	233.9	605.1				*Bcfpr2*	FK506-binding protein 2
Bcin03g05990.1	691.9	950.2	819.5				*Bccnb1*	Calcineurin subunit B
Bcin09g02390.1	287.1	131.4	566.7				*Bmp3*	Mitogen-activated protein kinase spm1
Bcin10g04140.1	310.6	426.5	183.9					Dual specificity protein kinase lkh1
Bcin11g02950.1	81.5	559.7	96.5					Serine/threonine-protein kinase prp4
Bcin11g04070.1	301.1	310.1	89.1					Calcium-transporting ATPase 2
Bcin14g03860.1	91.0	249.9	215.5	391.0	714.4	695.8	*Bcime2*	Sporulation protein kinase pit1
Bcin15g03580.1	403.5	69.3	59.7				*Bcsak1*	Mitogen-activated protein kinase hog1
Bcin16g01130.1	377.5	172.8	149.1				*Bcpka1*	cAMP-dependent protein kinase type 2
*Protein biosynthesis and regulation*								
Bcin02g06250.1	824.6	377.5	325.6					Tubulin-folding cofactor C
Bcin02g06900.1	716.6	2952	848.8				*Bcrps20*	40S ribosomal protein S20
Bcin03g05960.1	2454	842.8	2180.7				*Bcgst25*	Elongation factor 1-gamma 1
Bcin03g06970.1				4723	10 790	4203		40S ribosomal protein S14
Bcin07g01170.1	3936	2703	4662	4228	5795	5644		Eukaryotic translation initiation factor 5A
Bcin12g00420.1	444.3	305.1	1052				*Bcnmd3*	60S ribosomal export protein NMD3
Bcin12g03890.1	178.2	489.4	211.0					Exocyst complex component exo84
*Carbohydrate metabolism*								
Bcin01g06740.1	97.8	268.7	115.9					Efflux pump radE
Bcin01g07270.1	230.9	317.1	547.0				*Bchxt19*	Probable quinate permease
Bcin01g09950.1				442.4	1616	1968		Pyruvate carboxylase
Bcin01g10310.1	330.2	113.4	97.8				*Bcgdb1*	Glycogen debranching enzyme
Bcin02g01650.1	492.3	225.3	194.4					Maltose permease MAL31
Bcin02g08340.1	538.8	739.9	638.1	771.6	705.0	686.6	*Bctps1*	Alpha,alpha-trehalose-phosphate synthase [UDP-forming] 1
Bcin07g00940.3	112.7	51.6	44.5				*Bctps2*	Trehalose-phosphatase
Bcin08g00740.1	649.5	446.0	769.3					MFS glucose transporter mfs1
Bcin09g00150.1	415.3	380.2	983.9				*Bchex1*	High-affinity fructose transporter ght6
Bcin10g01500.1				1544	1411	1374		ATP synthase subunit 9, mitochondrial
Bcin11g05700.1	835.3	382.4	989.3				*Bchxk*	Hexokinase
Bcin12g02300.1	986.4	1083	467.3					Probable glucose transporter rco-3
Bcin15g02270.1	680.3	373.7	805.8	1169	1068	1560		Glycogen [starch] synthase
Bcin15g03620.1	229.8	473.4	136.1				*Bcgph1*	Glycogen phosphorylase
*Lipid metabolism*								
Bcin01g00440.1	212.0	194.1	83.7				*Bcfas2*	Fatty acid synthase subunit alpha
Bcin02g00210.1				1866	1705	553.6		Lipase 1
Bcin04g00760.1	301.2	413.7	178.4				*Bcfaa2*	Long chain acyl-CoA synthetase 7, peroxisomal
Bcin04g01780.1				894.6	817.3	2388	*Bcach1*	Acetyl-CoA hydrolase
Bcin07g06960.1	291.3	120.0	172.5					Acetyl-CoA carboxylase
Bcin09g02790.1	480.4	659.7	948.3	688.0	3143	3061		Acetyl-coenzyme A synthetase
Bcin16g03180.1	261.8	1078	1240					Acyl-CoA dehydrogenase family member 11
*Cell Wall metabolism*								
Bcin01g03390.1	62.6	85.9	222.3	268.8	245.6	478.5	*Bcams1*	Alpha-mannosidase
Bcin01g03790.2	46.5	63.9	55.1				*BcCHSIV*	Chitin synthase 4
Bcin02g06930.1				555.1	760.8	247.0		1,3-beta-glucan synthase component FKS1
Bcin03g00640.1	1904	1664	205.1					Alcohol oxidase 1
Bcin04g01290.1	968.6	532.0	229.4					Mannan polymerase II complex anp1 subunit
Bcin04g03120.1	561.4	154.2	531.9				*BcCHSIIIa*	Chitin synthase G
Bcin06g07010.1	1540	705.0	3648					Acetylxylan esterase
Bcin07g04810.1	91.9	126.2	108.8					Beta-hexosaminidase
Bcin08g00910.1	578.6	397.3	342.7				*Bcbgl2*	Glucan 1,3-beta-glucosidase
Bcin08g02140.1	162.7	148.9	385.4					Cell wall alpha-1,3-glucan synthase ags1
Bcin09g01110.1	1051	206.3	1245.4	645.4	1769	574.3		Exoglucanase 1
Bcin10g02280.1	563.6	193.5	834.5					Glycogenin-1
Bcin10g05590.1	218.6	100.1	86.3					Beta-glucosidase A
Bcin10g06130.1	461.6	633.9	273.4					Alpha-L-rhamnosidase rgxB
Bcin11g04800.1				1104	2017	1965		Chitin deacetylase ARB_04768
Bcin12g05360.1				616.7	563.5	823.1	*BcCHSVI*	Chitin synthase 6
Bcin13g04610.1	99.7	136.9	118.1					Sterol 3-beta-glucosyltransferase UGT80A2
Bcin15g00810.1	458.3	1048	542.8	656.3	599.6	2920		Cell wall integrity and stress response component 3

Additionally, many genes associated with fungal cellular degradation processes (e.g. autophagy, proteases, and ubiquitin-dependent protein catabolic process) and fungal cell wall organization (such as several carbohydrate-active enzymes (CAZymes) and chitin synthases) were detected as expressed in Syrah but not in Trincadeira (Fig. 5 and Supplementary Table S7). CAZymes allow plant tissue colonization through host-cell wall modifications and release of carbohydrates for fungus consumption [43, 44]. In detail, 46 and 16 annotated CAZymes were detected as expressed by *Botrytis cinerea* in Syrah and Trincadeira, respectively (Table 2 and Supplementary Table S7). Interestingly, several genes with a putative role in carbohydrate conversion were expressed mostly in Syrah berries. This includes, in particular, genes related to glycogen metabolism, glycolytic processes, TCA cycle, and trehalose biosynthesis (Table 2 and Supplementary Table S7). Fungal energy metabolism was also activated in the tolerant cultivar, as suggested by the expression of genes related to ATP synthesis and ATPase activity. Finally, several genes encoding players of lipid and fatty acid metabolism were also mainly expressed in Syrah (Table 2 and Supplementary Table S7) together with fungal major facilitator superfamily (MFS) and sugar transporters. The expression of genes encoding ABC transporters as well as genes involved in the glyoxylate cycle were noticed in both infected grapes (Table 2 and Supplementary Table S7).

On the other hand, some virulence-related fungal genes were also detected in Trincadeira infected berries despite their more advanced state of infection (Fig. 1 and Table 2), namely genes associated with sexual reproduction, fruit body formation, sporulation, and host colonization (Table 2). Moreover, a precursor of riboflavin, lipase 1, and a chitin deacetylase were identified only in Trincadeira and were described as important for *B. cinerea* infection strategy [25, 45].

## Discussion

Gray mold is one of the most problematic diseases affecting grapevines [46] and, even though recent studies focus on the molecular basis of *B. cinerea* pathogenicity [22–24], the processes behind necrotrophic infection of fruits at early ripening stages remain uncharted. Moreover, the combined analysis of both susceptible and tolerant green berries towards *Botrytis* infection has not been performed previously, leaving a gap in the knowledge of the complex and temporal dynamics of *V. vinifera/B. cinerea* pathosystem. Previous to the present study, mechanisms involved in susceptibility of Trincadeira berries were analyzed considering omics approaches [28]. Late green (EL33) and veraison (EL35) berries with grey mold symptoms evidenced a reprogramming of carbohydrate and lipid metabolisms with a putative involvement of jasmonic acid, ethylene, polyamines, and auxins [28]. In this study, we confirmed that this metabolic reprogramming occurs even at an earlier stage of berry ripening (EL32) in the susceptible variety. In contrast, the tolerant Syrah variety remained largely unaffected at early stages. Analysis of the fungal transcriptome indicates that *B. cinerea* is in a more virulent stage of interaction with the tolerant variety, revealing putative new mechanisms associated with this fungal infection.

### Pre-activated defenses in Syrah are likely to be responsible for its resilience against Botrytis cinerea attack

Transcriptome and metabolome analyses revealed that Syrah metabolism was only slightly modulated by *Botrytis cinerea* infection, suggesting that tolerance is mainly due to basal defenses. Interestingly, and even though no genes for complete resistance (R genes) to *B. cinerea* have been recognized in plants, functional category enrichment analysis revealed that R proteins and protein kinases were constitutively upregulated in Syrah, while their expression was triggered in Trincadeira by infection. The same was true for genes related to Ca^2+^ mediated signaling, JA signaling pathway, and ET biosynthesis. Calcium signaling modulates the regulation of protein kinases, and SA, ET, and JA metabolism [47], which participate in plant response to *B. cinerea* infection [16, 25, 28] and might likewise be important for basal tolerance. Furthermore, plasma membrane Ca^2+^ ATPases appear to be important components of receptor-mediated signaling for plant immune responses and development [48, 49].

The regulation of transcription is known to be paramount for an effective plant defense [50]. Several genes coding for transcription factors (ZIM, JAZ, ERF, AP2, WRKY, NAC) were highly expressed in Syrah healthy berries and upregulated in Trincadeira under infection. Orthologous genes of three transcription factors (*WRKY33*, *BOS1*, and *MIC2*) that influence immune responses in *A. thaliana* [51–53] were also noticed in this interaction and might contribute to the basal resistance of Syrah. The same holds true for genes belonging to the GRAS family of transcription factors involved in plant response to biotic stress [38].

Omics data underscored a distinct reprogramming of metabolic pathways between cultivars. As previously reported for a more advanced green stage, EL33 [28], the primary metabolism was activated in Trincadeira in response to *Botrytis* infection albeit photosynthesis appeared to be inhibited. This is a typical response to biotic stress, putatively compensating for activation of defense-related pathways and/or feedback regulation mediated by sugar signals [54] (reviewed by Rojas et al. [54]). Additionally, the expression of genes involved in carbohydrate metabolism and also fatty acid metabolism are known to affect downstream defense responses against fungal pathogens [15, 28, 54]. Transcript and metabolite analyses also indicated that Syrah may better cope with oxidative stress induced by *B. cinerea,* namely due to accumulation of the antioxidant α-tocopherol. In fact, a recent study showed that the absence of α-tocopherol in *A. thaliana* leaf chloroplasts may delay plant defense activation against *B. cinerea* through enhanced lipid peroxidation [55].

Transcriptomic data showed that Trincadeira green berries respond to infection by up-regulating genes involved in lipid metabolism (e.g. α-linolenic acid metabolism). Interestingly, high content in long-chain saturated hexacosanoic acid (cerotic acid) and of a triterpenoid of the ursolic/ oleanolic acid family were observed in Syrah at basal level when compared to Trincadeira. Moreover, genes involved in triterpenoid biosynthesis (coding for β-amyrin synthases) were upregulated in Syrah at basal level. Therefore, the basal tolerance observed in Syrah may rely on the pre-activated lipid-related defenses. Ursolic and oleanolic acids are commonly found in epicuticular waxes of plants and in grapes in particular [56]. These compounds also showed antifungal properties in apple [57] and very-long-chain fatty acids, such as hexacosanoic acid have been associated with plant defense [31].

Many genes involved in phenylpropanoid, and flavonoid pathways were upregulated in Syrah at basal level (Fig. 4 and Table 1). Metabolomic data showed a higher constitutive presence of the phenylpropanoids *trans*-4-hydroxycinnamic acid and epigallocatechin in Syrah (Fig. 2 and Fig. 3). Epigallocatechin is a precursor of epigallocatechin-3-gallate that is known for its antioxidant properties and has been suggested to promote jasmonic acid signaling in *A. thaliana*, increasing the resistance to *B. cinerea* [58].

The putative and positive metabolic markers involved in Syrah basal tolerance also included the volatiles 2-ethylfuran, hexanal, and (E, E)-2,4-Hexadien-1-al. Although studies addressing the role of plant volatiles during necrotrophic infection are scarce, Utto and colleagues (2008) showed that hexanal reduces postharvest infection of tomatoes by *B. cinerea* [59]*.* Also, 2-ethylfuran has been reported to prevent downy mildew symptoms in grapevine leaves [60]. Volatiles are indeed involved in resistance to fungal pathogens and they can even contribute to resistance-related phenotypes of neighboring receiver plants [61, 62]. Furthermore, the volatiles benzaldehyde and decanal were accumulated in Trincadeira upon infection and might be used, once validated, as markers of an advanced *B. cinerea* infection stage.

### Successful defense in Syrah putatively induces wide activation of specific signaling pathways and carbohydrate metabolism in Botrytis cinerea

The *Botrytis cinerea* transcriptome *in planta was* addressed in a few species, such as *A. thaliana*^6^, cucumber [21], kiwifruit [22], tomato, and others [23]. However, the molecular mechanisms associated with successful *B. cinerea* infection during the early stages of fruit ripening are unknown. In grapevine, the fungal transcriptome was explored at flowering stage [18], berry mature stage [24, 25], and fungal quiescence on hard green berries [25]. Haile and colleagues (2020) proposed a basal metabolic activity during quiescence with only 289 fungal genes expressed in hard green berries in contrast with the transcriptional reprogramming observed in this study [25] (Supplementary Table S2).

**Figure 6:**
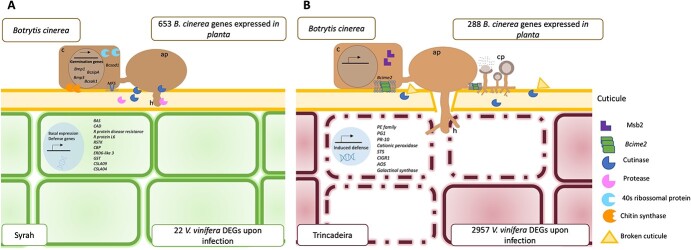
General representation of plant colonization by *Botrytis cinerea* in tolerant and susceptible cultivars (A) Syrah’s photosynthetic tissues are shown in green and represent healthy and green tissues with activated basal defenses (largely unchanged by the infection) namely the expression of genes coding for R proteins and secondary metabolism related. On the other hand, the fungus enters an active virulence program involving among others the release of cutinases and proteases; (B) Trincadeira’s photosynthetic tissues are shown in bordeaux color and represent infected cells with defenses largely activated by fungal presence namely the expression of genes involved in cell wall, hormonal, primary and secondary metabolisms. Opposite behavior is noticed in the fungus by presenting limiting transcriptional activity but related with a more advanced stage of infection namely the expression of genes involved in sporulation. Solid green or bordeaux lines represent living tissue, and dashed lines represent dead tissue. Fungal and plant cells are not proportionally scaled. Ap, appressorium; C, conidia. Plant genes: *Beta amyrin synthase –BAS; Cinnamyl alcohol dehydrogenase – CAD; R protein disease resistance; R protein L6; Sugar transporter ERD6 like 3- ERD6 like 3; Glutathione S-transferase 25–GST; Receptor serine/threonine kinase –RSTK; Calmodulin binding protein –CBP; Cellulose synthase CSLA09–CSLA09; Cellulose synthase CSLA04- CSLA04; Pectinesterase family –PE family;* Polygalacturonase - *PG1; Pathogenesis protein 10–PR-10; Cationic peroxidase; Stilbene synthase –STS; Chitin-inducible gibberellin-responsive protein 1–CIGR1; Allene oxide synthase- AOS*; *Galactinol synthase.* Fungal genes: *mitogen-activated protein kinase- mp1; transcription factor MBZ1- BczipA; Mitogen activated protein kinase spm1- Bmp3; Mitogen activated protein kinase hog1- Bcsak1; Superoxide dismutase [Cu-Zn]- Bcsod1; MFS transporter ptIL- MFS; Sporulation protein kinase pit- Bcime2; cutinase gene palindrome- binding protein- Bcwcl2; Pro-apoptotic serine protease NMA111- Bcnma; signaling mucin MSB2- Bcmsb2.*

In general, green fruits are reported as being resistant to infection by *B. cinerea,* which remains quiescent until the onset of ripening*.* However, we previously reported green berries of the highly susceptible cultivar Trincadeira exhibiting heavy symptoms of infection [16, 28] In this study, we confirmed that the fungus is active in Trincadeira but, more surprisingly, it seems highly virulent and far from quiescent in the Syrah cultivar (Fig. 6). From the 653 *B. cinerea* genes expressed in Syrah green berries, many were virulence-related (Table 2, Supplemental Tables S7-S8) and only seven genes were in common with the quiescent state described by Haille et al. [25] (Supplementary Table S9).

Moreover, transcriptomic analysis showed a ratio of 13/1000 and 57/1000 of *B. cinerea* to *V. vinifera* reads in Trincadeira and Syrah respectively, indicating a higher transcriptional effort of the necrotrophic fungus to proliferate and overcome Syrah’s basal defenses during infection. The abundance of pathogen transcripts seems to be partially related to fungal biomass and virulence [6, 25, 44]. Our results support the presence of a highly virulent fungus in green fruits of the tolerant cultivar; such high virulence might be related to the natural variation of pathogen strains [41, 43]. Moreover, in Trincadeira green berries only 288 fungal genes were detected, even though symptoms of infection were clear (Fig. 1D, Fig. 6). It can be expected that fungal virulence in this cultivar was also higher before the development of symptoms indicating that the fungus may lose virulence-associated mechanisms when the infection was successful. Similar temporal transcriptional dynamics were observed in the white-rot fungus *Obba rivulosa,* where a higher level of virulence associated gene expression was detected at early stages of wood colonization, after which the majority of those genes revealed reduced expression [28, 63].

Among the *Botrytis cinerea* virulence- and growth-related genes expressed in Syrah were noticed chitin synthases (*BcCHSIIIa and BcCHSIV*), genes involved in germination (*Bcg3, Bac* and *Bmp1*), conidia regulators (*Bmp3 and Bcsak1*), resistance to cyclosporin A *(Bcp1)*^43^ and elicitors of hypersensitive response such as *BcSpl1* [64]. Moreover, many genes putatively associated with signaling (e.g. protein phosphorylation and calcium-mediated) and transcription factors were expressed only in Syrah and might be novel and important elements of pathogenesis. Additionally, the expression in Syrah of many genes involved in chromatin structure and modification reenforce the putative association between epigenetic mechanisms and virulence, as previously reported [65].

The fungal transcriptome results suggested that transcriptional activity and protein synthesis were activated during infection especially in Syrah green berries, including the expression of several genes coding for regulators of transcription, ribosomal related, protein folding and protein phosphorylation. Such profile was observed during a quiescent *Botrytis cinerea* infection and in response to phytoalexins in grapevine [25, 66]. Plants typically trigger an oxidative burst at the early stages of infection, generating several ROS to counteract pathogen invasion. However, as a necrotrophic fungus, *B. cinerea* can take advantage of that and even produce its own ROS [67]. Several fungal key players in oxidative stress were expressed mainly in Syrah, such as *BcNoxR* essential for virulence [67], the generator of H_2_O_2_ (*Bcsod1*), peroxidases, and others. Interestingly, polyamine metabolism, which is associated with responses to stresses and ROS scavenging was also activated in the fungus infecting Syrah (*Bcin14g01740.1*, *Bcin11g03520.1*, *Bctpo5,* and *Bcspe2*) and might have novel roles during *B. cinerea* infection [68].

Fungal necrotrophic infection also relies on the secretion of enzymes to exploit and disassemble cell wall polysaccharides and use them as the main energy source [21]. Actually, several CAZymes involved in such processes were expressed by the fungus mainly in Syrah with glycosyltransferases and glycoside hydrolases being the most representative classes. Moreover, the expression of ABC and MFS transporters, together with several genes putatively encoding peptidases in Syrah might suggest an attempt of the fungus to mitigate the action of host defense. Indeed, in *A. thaliana, B. cinerea* ABC transporters were shown to be essential for tolerance against camalexin whereas MFS transporters provided tolerance to glucosinolate-breakdown products and were required for pathogenicity [69, 70]. Furthermore, genes involved in fungal exocytosis (e.g. vesicle-mediated transport) were also expressed mainly in Syrah; such active transport is crucial for virulence and typically involved in the growth or exudation of fungal toxins to the intercellular space [6]. Moreover, genes involved in autophagy processes were expressed in Syrah and might have an important role during early infection characterized by nutritional limitations, since it is a process of recycling unnecessary or dysfunctional cellular components with great influence on conidial germination and virulence [71, 72].

During infection, the fungus converts plant hexoses and fructose into mannitol, which together with trehalose, are the most common fungal storage carbohydrates. Interestingly, fungal carbohydrate metabolism was active in Syrah, as suggested by the expression of genes involved in glycogen metabolism, glycolytic processes, and trehalose biosynthesis. Similar results were described during sunflower infection [73] and suggests the targeting of glucose into the TCA cycle. Moreover, fungal lipid and fatty acid metabolisms appear to be active *in planta* during interaction with the tolerant cultivar, as suggested by the expression of genes coding for of acetyl-CoA dehydrogenase and carboxylases, long-chain acyl-CoA synthetase 7, and others. The association between lipid and carbon metabolisms involving glyoxylate and TCA cycles was previously hypothesized as fundamental for early fungus development and host invasion before having access to host nutrients [74]. On the other hand, several genes associated with gluconeogenesis (e.g. pyruvate carboxylase, a pyruvate kinase, two glucose-6-phosphate, and others) were expressed only in Trincadeira and are likely associated with fungal proliferation after successful infection. Since *B. cinerea* was only capable of successful infection in Trincadeira, these results may be important to understand the dynamics of proliferation and infection strategies of the fungus. In particular, they provide insights into how fungal and plant carbohydrate metabolisms are balanced with both fungal and plant defensive strategies.

A comparative meta-analysis was performed in order to retrieve differences between our data and the *Botrytis cinerea* transcriptome during infection of ripening berries as described by Haile et al. (2020) [25]. This study integrated transcriptome and metabolome data to investigate the crosstalk between the plant and the fungus during pathogen quiescence and egression. It is noteworthy to mention the limitations of comparing both studies since different pathogenic infection stages were considered. Nevertheless, the comparison revealed a broad repertoire of expressed transcripts (Supplemental Table S9), which is indicative of high genome plasticity and transcriptional flexibility [6, 67, 75]. In fact, this plasticity may contribute to the aptitude of *Botrytis cinerea* to infect a wide number of plant species. Common functional classes were also observed among the expressed genes, such as redox processes, ATP-related, or protein folding classes. In both studies, several genes coding for ribosomal proteins were also expressed suggesting that ribosomes and translation may play a fundamental function in the infection process. Plant ribosomal proteins have been recently associated with biotic stress responses [76], but their role in pathogenesis remains intriguing. Moreover, an uncharacterized secreted protein *(Bcin15g00100)* was expressed in all the samples analyzed in both studies, deserving special attention since the orthologous in *Blumeria graminis* was associated with virulence [77].

## Conclusions

Understanding grey mold disease processes may disclose new and efficient management strategies. Our study revealed a contrasting response between Syrah and Trincadeira cultivars, underlining the importance of studying cultivars with different susceptibility/tolerance levels and specifically at a stage that is generally thought be low or even non-susceptible. Syrah was barely affected by *B. cinerea* infection at the green stage, eventually due to pre-activated defensive mechanisms involving specific signaling pathways, hormonal regulation and secondary metabolism. In contrast, Trincadeira was severely affected by *B. cinerea* and reprogrammed primary and secondary metabolisms, putatively regulated by jasmonate- and ethylene- mediated signaling pathways and transcription factors of the ZIM/JAZ, NAC, MYB, ERF and GRAS families. This study also suggested promising metabolic markers of tolerance against grey mold disease at early stages, including 2-ethylfuran, hexanal, (E, E)-2,4-Hexadien-1-al, cinnamic acid, shikimic acid, hexacosanoic acid, phenylacetaldehyde, and epigallocatechin.

An opposite scenario was found in the fungus with higher transcriptional activity shown in the tolerant cultivar. The study put in evidence the plasticity of the pathogen’s transcriptome, revealing several genes related to virulence and fungal growth, signaling, carbohydrate and lipid metabolism expressed during infection of Syrah green berries, which might be important for early stages of infection. Nevertheless, different genes related to growth and virulence were also detected only in Trincadeira and might be important to understand regulatory mechanisms behind necrotrophic fungus proliferation after successful infection. Since only few genes have been described previously to be involved in pathogenicity [67], the newly identified putative elements of virulence might be targeted for functional characterization and to develop efficient control strategies. *Botrytis cinerea* infects several plant species worldwide and the knowledge gathered in this *in vivo* pathogen interaction study may provide valuable hints to be translated to other plant species.

## Materials & methods

### Plant material and fruit inoculation

Contaminated grapevine plants were the source to isolate *B. cinerea* which was maintained at 5°C in potato dextrose agar (Difco, Detroit, MI, USA). Green berry clusters (Trincadeira and Syrah) were sprayed with a conidial suspension EL29 [28] and collected after visual monitoring of symptoms at green stage EL32 (modified E–L system [30]). Control clusters were sprayed with phosphate buffer. Samples were shortly placed on ice, frozen in liquid nitrogen, and kept at −80°C until further use. Preceding extraction for transcriptomics and metabolomics, seeds were eliminated and samples grinded in liquid nitrogen. Three-four biological replicates of Syrah and Trincadeira green berries (control and infected) were considered for RNA-sequencing and metabolomics.

### RNA extraction, sequencing, and gene expression analysis

RNA was extracted as mentioned in Fortes et al. [78] with modifications [16]. RNA quantity and integrity were evaluated as previously [16]. Sequencing was performed at the Centre for Genomic Regulation (Barcelona). TruSeq Stranded mRNA Sample Prep Kit v2 (ref. RS-122-2101/2) was used to prepare the libraries. Libraries were sequenced (50 nt paired-end) on the Illumina HiSeq 2500 using v4 chemistry. Twelve libraries were sequenced for Syrah and Trincadeira samples (6 control and 6 infected). Illumina raw read data was placed in the NCBI Sequence Read Archive (SRA; PRJNA611792). Raw reads generated were checked for general quality and presence of adapters or contaminants via FastQC analysis [79]. Quality trimming and filtering of raw reads were done with an in-house script with a threshold of 30 (quality score). Ten nucleotides at 5′-end were trimmed from each sequence of all libraries.

Grapevine (12Xv1, http://genomes.cribi.unipd.it/) and *B. cinerea* (strain B05.10) (ASM83294v1, http://fungi.ensembl.org) were the selected reference genomes. HISAT2 (v. 2.1.0) aligned processed reads to the combined references to identify the splice sites independently of the annotations [80]. The mapping parameters were as follows: —rdg 2,2 —rfg2,2 — mp4,2 —rna-strandness RF. The software package SAMtools (v. 1.3.1) (http://samtools.sourceforge.net/) was used for processing of the mapped reads, such as removal of duplicates (rmdup). The HTSeq tool (version0.11.1) counted the strand-specific read-pairs mapped to the exon regions annotated in the grapevine (12Xv1) and *B. cinerea* (ASM83294v1) genomes [81]. Differential gene expression analysis was performed using the Bioconductor package EdgeR (v. 3.24.2) [82]. Total read counts were first normalized by library size using the trimmed means of median (TMM) method [82]. Normalization of depth and gene length were done by transforming pair-read to fragments per kb per million counts (RPKM). For differential expression analysis a RPKM >10 threshold was used; dispersion among samples was evaluated, and an ANOVA-like test was conducted for any pairwise comparison with exactTest function.

Differentially expressed grapevine genes (DEGs) were significantly changed at the FDR ≤ 0.05 and |log_2_ fold change (log_2_FC)| > 1.0. *B. cinerea* genes were handled as present or absent, comparing infected vs. control samples for both cultivars. Genes were only considered present if normalized gene count was equal to zero in all three control replicates and higher than twenty in all three infected replicates. Moreover, *B. cinerea* genes present in Syrah and absent in Trincadeira were described as Syrah-specific, and *vice versa.*

### Functional enrichment analysis

Significant functional enrichment in *V. vinifera* DEGs was identified with FatiGO [35] and by using a grapevine-specific functional classification of 12X V1 genome assembly predicted genes [83]. Fisher’s exact test was performed to compare these outputs with the list of total non-redundant genes in the grapevine genome. Enrichment was significant for P-value ≤0.05 following Benjamini and Hochberg correction for multiple testing. Transcripts of *B. cinerea* were functional annotated according to [5] which was manually updated by literature review.

### Soluble metabolites

Soluble metabolite profiling was performed by using Gas chromatography coupled to electron impact ionization time-of-flight mass spectrometry (GC-EI/TOF-MS) [84]. Soluble metabolites were extracted from deep-frozen powder in ethyl acetate for 2 h agitation at 30°C [85]. After centrifugation, two aliquots from the ethyl acetate fraction were dried by vacuum concentration and kept at −20°C.

Chemical derivatization and retention index calibration were conducted before injection [84]. GC-EI/TOF-MS analysis was performed using an Agilent 6890 N24 gas chromatograph (Agilent Technologies, Germany) connected to a Pegasus III time-of-flight mass spectrometer (LECO Instrument GmbH, Germany) [28]. Chromatograms were processed as previously detailed [28].

Identification of compounds was performed based on mass spectra and retention time index matching to Golm Metabolome Database [86, 87] and by using TagFinder software [88] (considering the presence of at least three specific mass fragments per compound and a retention index deviation of less than 1.0% [89]). Metabolite intensities were normalized with fresh weight and internal standard (C22), maximum scaled, and log_2_-transformed to approximate normal distribution. A subset of metabolites was identified only by mass spectral match as indicated by square brackets, e.g. [ursolic acid]. This compound differs in in retention index from expected [90] and identified abundant oleanolic acid (Supplemental Table S1) and matches best to ursolic acid. Due a deviation from the expected retention index and the presence of other pentacyclic triterpenoids [91] in *V. vinifera*, we annotated [ursolic acid] as a triterpenoid of the ursolic/ oleanoic acid family.

### Volatile metabolites

Volatile profiling (500 ± 50 mg fresh weight) was performed by solid-phase micro-extraction (SPME) and GC coupled to an electron impact ionization/quadrupole MS (GC-EI/QUAD-MS) using an Agilent 5975B VL GC–MSD system and according to *Vallarino* et al. [92]. SPME samples were removed from the headspace and processed as previously [28]. Programming for GC was 2 min isothermal at 40°C followed by a 10°C/min ramp to 260°C final temperature (held constant for 10 min). The Agilent 5975B VL GC–MSD system was operated with a continuous flow of helium at 1.0 mL/min. Desorption from the SPME fiber was at 16.6 psi with an initial 0.1 min pulsed-pressure at 25 psi. The subsequent purge was 1 min at a purge flow of 12.4 mL/min. System stability was controlled, and the sample sequence randomized. GC-EI/QUAD-MS chromatograms were acquired with the mass range set to 30–300 m/z and a 20 Hz scan rate. Chromatograms were obtained, visually controlled, and exported in NetCDF file format using Agilent ChemStation-Software (Agilent) and baseline-corrected with MetAlign software [93].

Compounds were identified matching to the reference collection of volatile compounds [86, 87] (presence of at least three specific mass fragments per compound and a retention time deviation of less than 3%). Metabolite intensities were then normalized by sample fresh weight, maximum scaled, and log_2_-transformed to approximate normal distribution.

### Statistical analysis

Metabolomics data (log2-transformed response ratios) was analyzed using Student’s t-test, one- and two-way ANOVA, Kruskal–Wallis, and Wilcoxon rank-sum tests. Benjamini & Hochberg correction was used for multiple comparisons, MetaGeneAlyse web application was used for principal component analysis (v.1.7.1; http://metagenealyse.mpimp-golm.mpg.de) and the R function prcomp to the log_2_-transformed response ratios with missing value substitution, log_2_ = 0. Heatmaps were obtained using the R package ComplexHeatmap [94]. Venny 2.1 web tool (https://bioinfogp.cnb.csic.es/tools/venny/) was used for Venn diagrams.

## Acknowledgments

Fundação para a Ciência e Tecnologia (FCT) supported the research through “GrapeInfectomics” (PTDC/ASP-HOR/28485/2017). This article/publication is based upon work from COST Action CA 17111 INTEGRAPE, supported by COST (European Cooperation in Science and Technology). FS and D.P. were recipients of fellowships from BioSys PhD programme PD65-2012 (PD/BD/114385/2016 and PB/BD/130976/2017, respectively).

## Author Contributions

A.M.F. designed and supervised the study; F.S., D.P., A.M.F., extracted RNA; D.P., A.E., J.K., performed metabolomics; P.R., C.R., performed infections; F.S., M.P., M.G.C. performed bioinformatic analysis of RNAseq; C. N. Performed one figure and assisted in tables and final draft; F.S. wrote the initial draft, completed by A.M.F.; M.G.C., J.K., A.M.F. revised the manuscript.

## Data availability

RNA-seq data is available at the NCBI SRA database under PRJNA611792 accession number.

## Conflict of interests

The authors declare no conflict of interest.

## Supplementary data

Supplementary data is available at *Horticulture Research* online.

## Supplementary Material

Web_Material_uhac217
