# Supplementary figures and images for: Virulence-related metabolism is activated in Botrytis cinerea mostly in the interaction with tolerant green grapes that remain largely unaffected in contrast with susceptible green grapes
Source: Hortic Res. 2022 Sep 21;9:uhac217. doi: 10.1093/hr/uhac217 (PMC9720446; doi:10.1093/hr/uhac217)

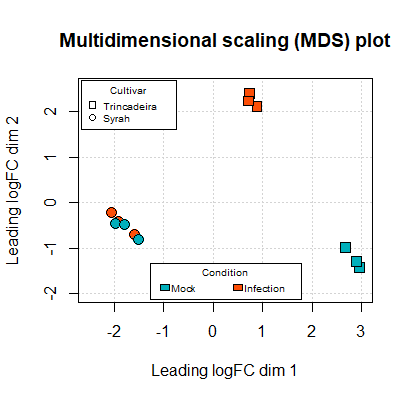

Supplement: Web_Material_uhac217 [file web_material_uhac217.zip › FigS2.tiff]

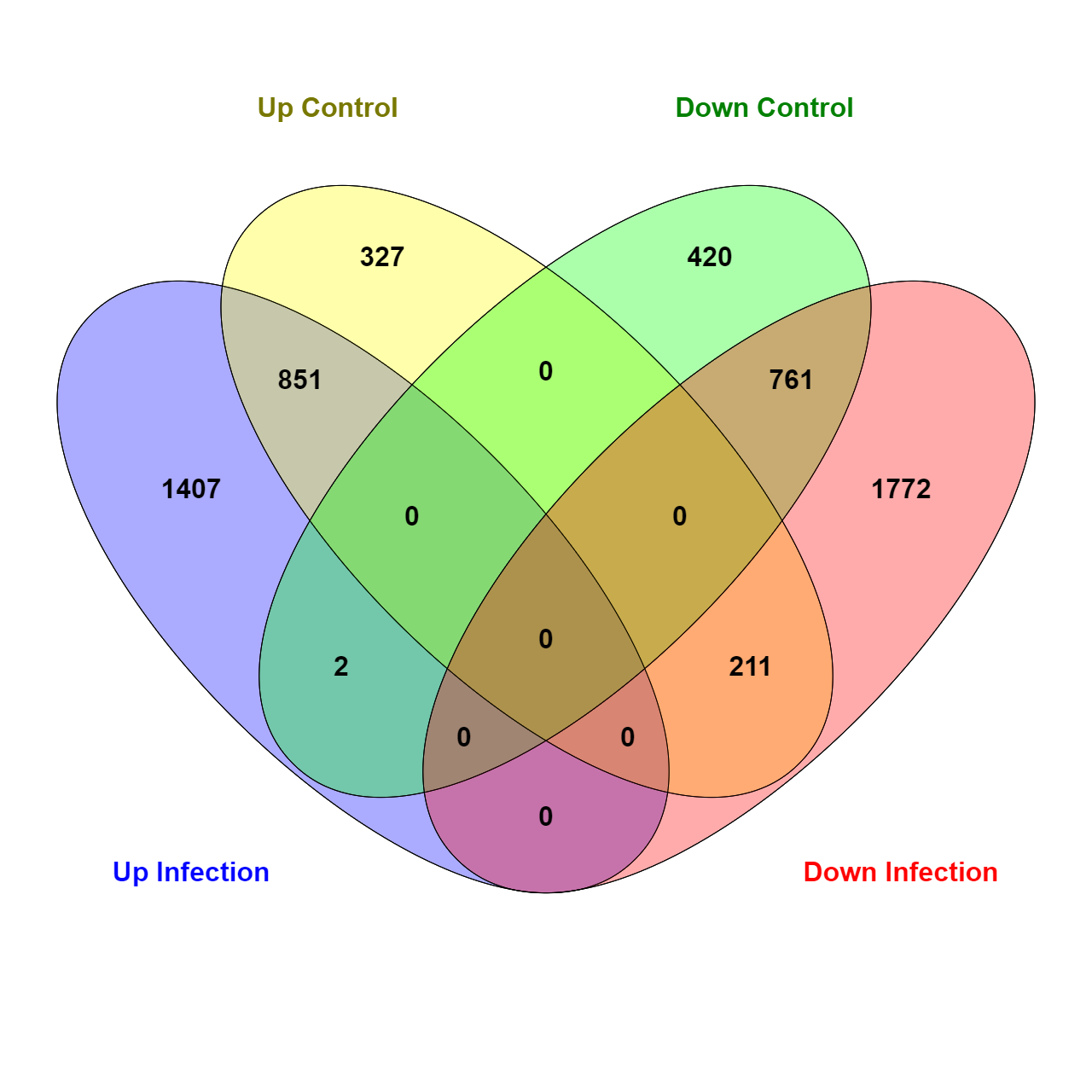

Supplement: Web_Material_uhac217 [file web_material_uhac217.zip › FigS3A.png]

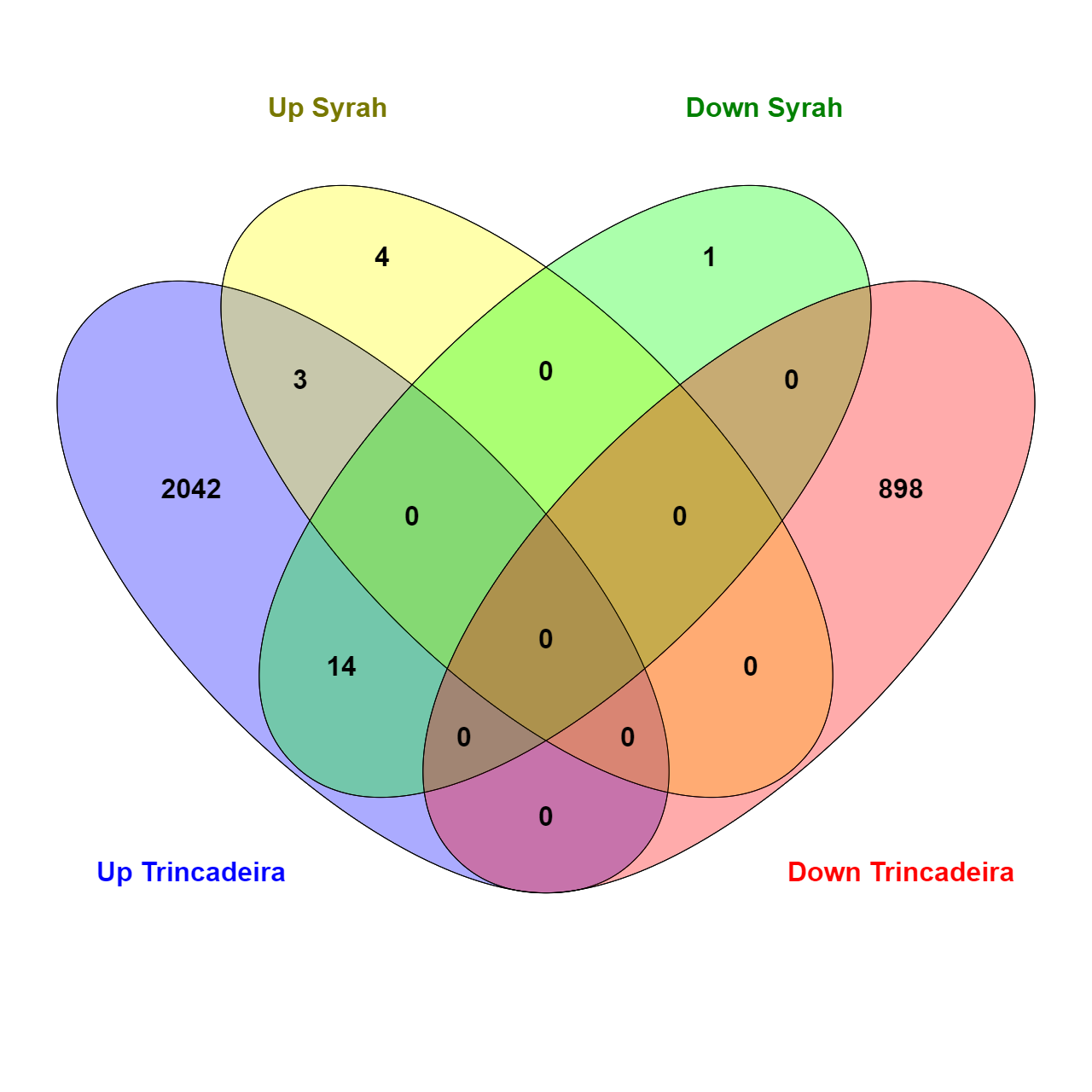

Supplement: Web_Material_uhac217 [file web_material_uhac217.zip › FigS3B.png]

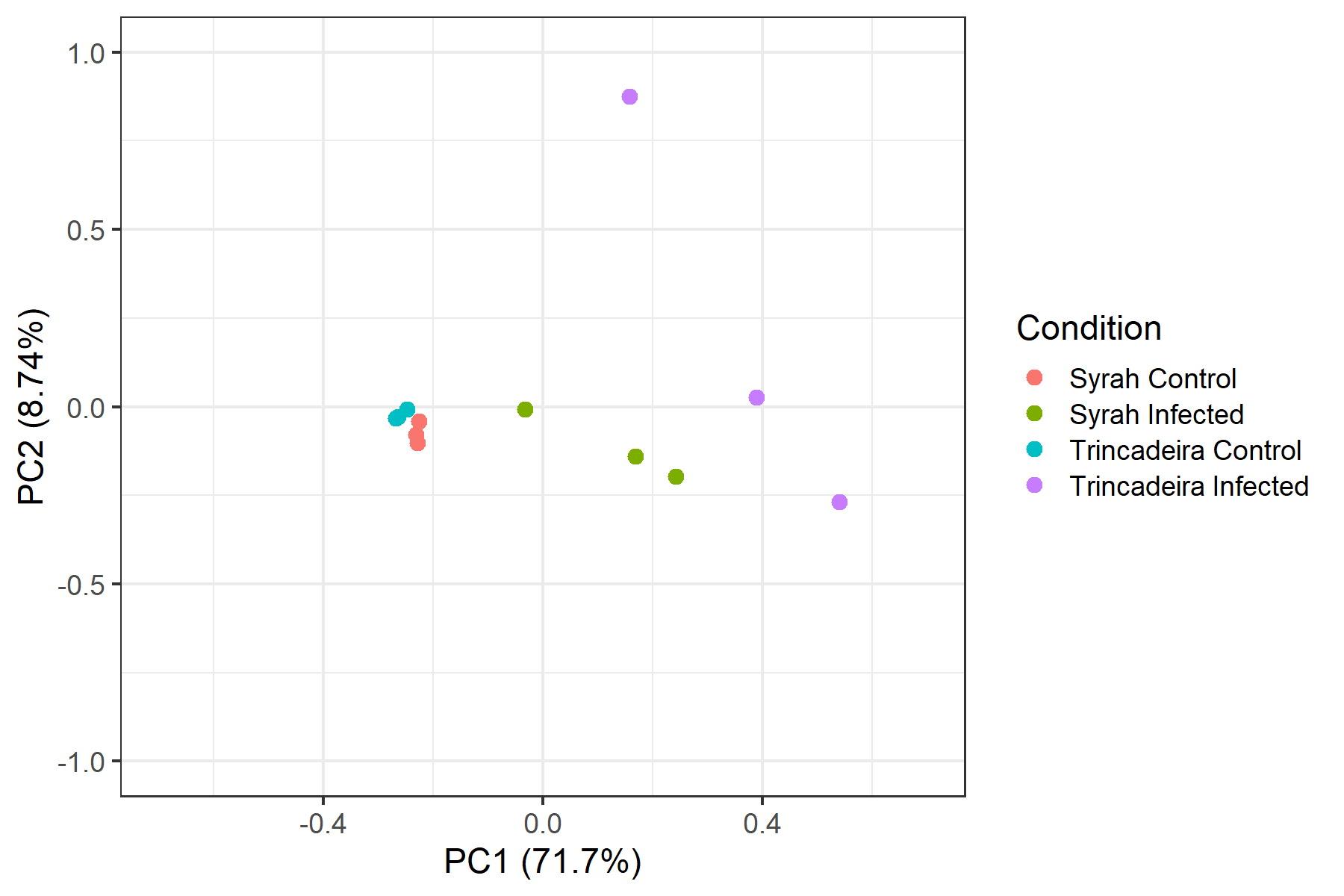

Supplement: Web_Material_uhac217 [file web_material_uhac217.zip › FigS4.png]
